# Defining genotype-phenotype relationships in patients with hypertrophic cardiomyopathy using cardiovascular magnetic resonance imaging

**DOI:** 10.1371/journal.pone.0217612

**Published:** 2019-06-14

**Authors:** Robert J. H. Miller, Shahriar Heidary, Aleksandra Pavlovic, Audrey Schlachter, Rajesh Dash, Dominik Fleischmann, Euan A. Ashley, Matthew T. Wheeler, Phillip C. Yang

**Affiliations:** 1 Division of Cardiovascular Medicine, Stanford University School of Medicine, Stanford, California, United States of America; 2 Center for Inherited Cardiovascular Disease, Division of Cardiovascular Medicine, Stanford University School of Medicine, Stanford, California, United States of America; 3 Department of Radiology, Stanford University School of Medicine, Stanford, California, United States of America; Scuola Superiore Sant'Anna, ITALY

## Abstract

**Purpose:**

HCM is the most common inherited cardiomyopathy. Historically, there has been poor correlation between genotype and phenotype. However, CMR has the potential to more accurately assess disease phenotype. We characterized phenotype with CMR in a cohort of patients with confirmed HCM and high prevalence of genetic testing.

**Methods:**

Patients with a diagnosis of HCM, who had undergone contrast-enhanced CMR were identified. Left ventricular mass index (LVMI) and volumes were measured from steady-state free precession sequences. Late gadolinium enhancement (LGE) was quantified using the full width, half maximum method. All patients were prospectively followed for the development of septal reduction therapy, arrhythmia or death.

**Results:**

We included 273 patients, mean age 51.2 ± 15.5, 62.9% male. Of those patients 202 (74.0%) underwent genetic testing with 90 pathogenic, likely pathogenic, or rare variants and 13 variants of uncertain significance identified. Median follow-up was 1138 days. Mean LVMI was 82.7 ± 30.6 and 145 patients had late gadolinium enhancement (LGE). Patients with beta-myosin heavy chain (*MYH7*) mutations had higher LV ejection fraction (68.8 vs 59.1, p<0.001) than those with cardiac myosin binding protein C (*MYBPC3*) mutations. Patients with *MYBPC3* mutations were more likely to have LVEF < 55% (29.7% vs 4.9%, p = 0.005) or receive a defibrillator than those with *MYH7* mutations (54.1% vs 26.8%, p = 0.020).

**Conclusions:**

We found that patients with *MYBPC3* mutations were more likely to have impaired ventricular function and may be more prone to arrhythmic events. Larger studies using CMR phenotyping may be capable of identifying additional characteristics associated with less frequent genetic causes of HCM.

## Introduction

Hypertrophic cardiomyopathy (HCM) is a common hereditary cardiac disorder with a prevalence of approximately 2 cases per 1000 persons.[1] It is caused by mutations in genes encoding sarcomere proteins, [2,3] with more than two dozen putative disease-associated genes identified. *MYH7* encoding the β-myosin heavy chain and *MYBPC3* encoding cardiac myosin-binding protein C are the most common genes harboring causative mutations.[4–6] HCM is a frequent cause of sudden cardiac death (SCD) in youth and a significant underlying pathology for cardiac morbidity and mortality in adults.[5] It is believed that myocardial fibrosis, a hallmark of HCM, contributes to the development of SCD, ventricular tachyarrhythmias, and congestive heart failure (CHF).[7–11]

Cardiovascular magnetic resonance imaging (CMR) has emerged as a valuable tool for assessing HCM through quantification of ventricular volumes, mass, and identification of myocardial fibrosis with late gadolinium enhancement (LGE) to assess SCD risk.[12–14] The volume and morphology of LGE have been associated with worse cardiovascular outcome, including higher incidence and recurrence of ventricular tachyarrhythmia, hospital admissions due to progressive CHF, and an independent predictor of all cause and cardiac mortality.[15–18] It has also been demonstrated that LGE is more common in patients with a positive genetic test as compared to those with a negative genetic test; however, HCM is known for marked pleiotropy for any specific phenotype. [19,20]

The purpose of this study was to determine whether CMR findings could identify specific genotype-phenotype relationships in HCM through measurement of ventricular volumes, mass and function or characterization of LGE. Furthermore, we assessed the associations between CMR characteristics, genetic diagnosis and adverse cardiovascular events.

## Methods

### Patient population

This study was a retrospective analysis of data acquired in consecutive patients with HCM who underwent contrast-enhanced CMR at Stanford Hospital and Clinics between December 2006 and December 2017. Patients were excluded if the diagnosis of HCM could not be confirmed (n = 83) or if CMR studies were performed at an outside institution and images were not available for interpretation (n = 3). The diagnosis of HCM was based on standard clinical criteria including all components of the history, physical examination, electrocardiography, echocardiography, and CMR.[21] Alternate diagnoses including aortic stenosis and infiltrative cardiomyopathies were excluded by experienced cardiologists with additional training in HCM. Patients with a history of myectomy or alcohol septal ablation prior to CMR were excluded. Patient demographics were collected from existing patient records.

Patients were offered genetic testing with patient consent through clinical care at the Stanford Center for Inherited Cardiovascular Diseases. Genetic testing was performed in 202 patients. Genetic testing was performed with exonic sequencing of at least 8 myofilament-encoding, HCM-susceptibility genes as part of commercially available HCM genetic tests during the period of study (Ambry Genetics, Aliso Viejo, CA; Correlagen Diagnostics, Inc., Waltham, MA; GeneDX, Gaithersburg, MD; Invitae Corp, San Francisco, CA; Laboratory for Molecular Medicine, Cambridge MA; PGx Testing, Garden Ridge, TX; Transgenomic Molecular Laboratory, Omaha, NE). Sequences were compared with the reference human genome and variants detailed by the genetic testing company. All reported variants were independently investigated by the multidisciplinary team which included dedicated genetic counsellors and scored according to the confidence with which they could be called disease-causing.[22] This was based on type of variant, position of variant, prior co-segregation data, and in the case of novel variants, tools based on conservation and constraint as described previously.[23] S1 Table summarizes the classification of genetic variants. Patients were classified as having no variant found or having either variant of uncertain significance (VUS) or disease-associated variants meeting classification types ‘likely pathogenic’ or ‘pathogenic’. Gene variants which occurred at a population frequency <1 in 10,000 were categorized as rare variants. Patients with more than one variant were classified according to the disease causing-variant as described above, if one was found. No patients had disease-causing variants identified in more than one gene. Patients without a disease-associated variant, but with rare VUS in either *MYH7* or *MYBPC3* were included with patients with disease-associated variants in the respective genes. Separate analyses were performed in which these patients were included with other patients having a VUS as a sensitivity analysis, results in S2 and S3 Tables. Characteristics of patients who did not undergo genetic testing are shown in S4 Table.

### Image acquisition and analysis

CMRs were ordered as part of routine clinical care, and were typically performed to better delineate anatomy or to determine extent of LGE. All CMR images were acquired on a 1.5-Tesla whole-body scanner (Signa, GE Healthcare, Milwaukee, WI) with the patient in a supine position using an 8-element phased-array radiofrequency coil with breath-holding and cardiac gating. Cine images of the LV in short and long axes were acquired using a steady-state free precession sequence (SSFP, TR 2.4–3.9, TE 0.9–2.0, slice thickness 8 mm). LGE images (segmented k-space inversion recovery sequence, TR 3.4–5.0, TE 1.1–1.5, TI 150–300, slice thickness 8 mm) were acquired throughout the entire LV starting at 10 min, after administration of 0.1–0.2 mmol/kg of gadolinium diethylenetriaminepentaacetic acid (Gd-DTPA, Magnevist, Schering AG, Germany). The inversion time was set to null the signal of normal myocardium after Gd-DTPA and was adjusted during the scan as necessary.

**Fig 1:**
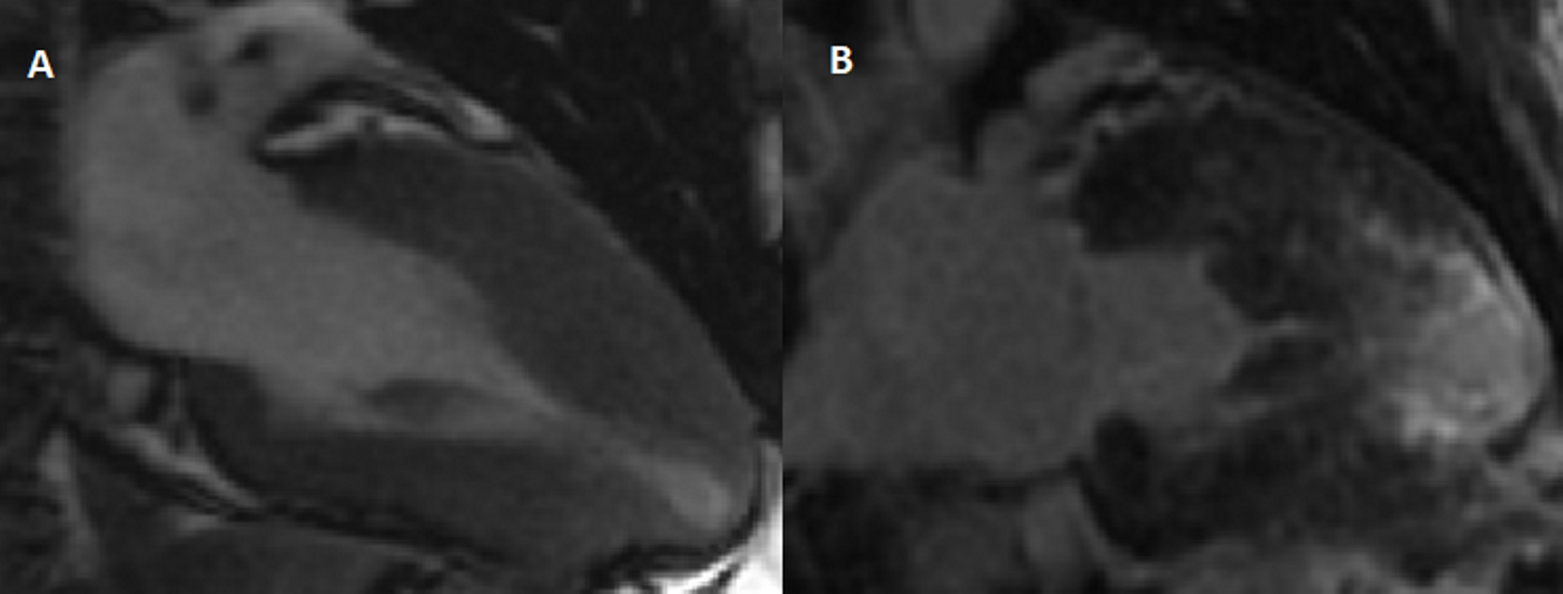
Cardiac magnetic resonance characterization of a patient with a *MYH7* variant. Panel A. Cine images at end systole showing apical hypertrophy with apical aneurysm. Panel B. Delayed enhancement images showing extensive LGE in the distal myocardial segments including the apex.

Cine images were analyzed using MASS analysis software (MASS Analysis Plus Version 6.0, Leiden University). Semi-automated contours were manually adjusted to match the endocardial and epicardial borders and exclude the papillary muscles from short-axis images at end-diastole.[24] The same contours were used to calculate left ventricular (LV) and right ventricular (RV) end-diastolic volumes (LVEDV and RVEDV), LV and RV end-systolic volumes (LVESV and RVESV), and LV and RV ejection fractions (LVEF and RVEF). Normal LV mass was defined as < 81 g/m^2^ for males and < 62 g/m^2^ for females.[24] Septal morphology and cavity contour was evaluated, in the long axis view and scored for 4 subtypes (sigmoid, reverse curvature, apical or other) as previously described.(26) Analysis of LGE was performed visually by defining the areas of hyperenhancement in all myocardial segments as seen on the long and short-axis slices and quantified using a full width, half maximum (FWHM) method. The FWHM method defines core scar as voxels that contain a signal intensity at least 50% of the maximal signal intensity.[25] Grey zone scar is defined as areas with less than 50% of the maximal intensity but greater than the peak signal intensity in remote myocardium.[25] A case example is shown in Fig 1. Two authors (RM and SH) performed image analysis. A subset of 10 patients was read by both authors for ventricular mass, volume, morphology, presence of LGE and scar quantification. Inter-rater reliability was good between readers (Pearson’s r>0.95 for all variables).

### Outcomes

Patients were followed prospectively for cardiovascular events including: alcohol septal ablation, septal myectomy, sustained VT, appropriate implanted cardiac defibrillator (ICD) shock, SCD, and all-cause death. SCD included patients with resuscitated cardiac arrest. Sustained VT was defined as ventricular rhythms faster than 100 beats per minute and lasting for more than 30 seconds or treated with anti-tachycardia pacing on review of ambulatory ECG monitoring or implanted cardiac device logs. Appropriate ICD shock was defined as a ventricular rhythm greater than 100 beats per minute which resulted in ICD discharge (excluding anti-tachycardia pacing). Follow up was obtained during scheduled clinic visits supplemented by telephone contact with patients or their relatives to ensure more complete follow-up. All patients had at least 90 days of clinical follow-up.

### Statistical analysis

Continuous variables were summarized as mean (standard deviation [SD]) if normally distributed and compared using a Student’s t-test. Continuous variables which were not normally distributed were summarized as median (interquartile range [IQR]) and compared using a Wilcoxon rank-sum test. Categorical variables are summarized as number (proportion) and compared using a Fisher’s Exact test.

We performed multivariable Cox regression analyses to assess for the association between the presence of late gadolinium enhancement and clinical outcomes as well as between genetic diagnoses and clinical outcomes. Due to low event numbers, events were combined as: septal ablation or myectomy, sustained VT or appropriate ICD shock, and all-cause mortality. Models were corrected for age and gender. All statistical tests were two-sided and a p-value <0.05 was considered significant. A sensitivity analysis with rare variants included as VUS, with results in S2 and S3 Tables. All analyses were performed using Stata/IC version 13.1 (StataCorp, College Station, Texas). The study protocol was approved by the Institutional Review Board at Stanford University.

## Results

### Clinical characteristics

We included 273 patients with a diagnosis of HCM who underwent CMR imaging. Patient characteristics are outlined in Table 1. The cohort was predominantly Caucasian (64.5%) men (62.9%) with a mean age 51.2 ± 15.5 (standard deviation). LGE was present in 145 (53.1%) patients. Patients with pathogenic, likely pathogenic or rare *MYH7* variants were a similar mean age as those with *MYBPC3* variants (45.7 vs. 45.9 years, p = 0.959). Patients with LGE were also less likely to be Caucasian (57.2% vs 72.7%, p = 0.011) and were more likely to be in New York Heart Association (NYHA) class I (65.5 vs 53.1%, p = 0.048).

**Table 1:** Baseline population characteristics.

	Total (n = 273)	No LGE (n = 128)	LGE+ (n = 145)	p-value
Age at CMR	51.2 ± 15.5	50.6 ± 15.0	52.0 ± 16.1	0.474
Male (%)	**173 (62.9)**	**73 (57.0)**	**100 (69.0)**	**0.045**
BSA (m2)	1.94 ± 0.25	1.95 ± 0.24	1.94 ± 0.26	0.859
Proband	268 (98.2)	125 (97.7)	143 (98.6)	0.668
Maximal LV wall thickness (mm)	**18 (16–21)**	**16 (15–19)**	**20 (16–24)**	**<0.001**
Ethnicity				
Caucasian	**176 (64.5)**	**93 (72.7)**	**83 (57.2)**	**0.011**
Asian	41 (15.0)	17 (13.3)	24 (16.6)	0.500
Latino	24 (8.8)	12 (9.4)	12 (8.3)	0.832
African-American	9 (3.3)	2 (1.6)	7 (4.8)	0.180
Other	**22 (8.1)**	**5 (3.9)**	**17 (11.7)**	**0.024**
Congestive Heart Failure				
NYHA I	**163 (59.7)**	**68 (53.1)**	**95 (65.5)**	**0.048**
NYHA II	73 (26.7)	37 (28.9)	36 (24.8)	0.494
NYHA III	34 (12.5)	21 (16.4)	13 (9.0)	0.069
NYHA IV	4 (1.5)	2 (1.6)	2 (1.4)	1.000
Resting LVOT gradient	2 (0–28)	4 (0–33)	1 (0–26)	0.574
Atrial Fibrillation	30 (11.0)	15 (11.7)	15 (10.3)	0.847
Dyslipidemia	84 (30.8)	37 (28.9)	47 (32.4)	0.600
Hypertension	108 (39.6)	57 (44.5)	51 (35.2)	0.137
Diabetes	19 (7.0)	6 (4.7)	13 (9.0)	0.233
Risk Factors for SCD				
LV wall thickness > 30 mm	**9 (3.3)**	**1 (0.8)**	**8 (5.5)**	**0.039**
FH SCD	108 (39.6)	44 (34.4)	64 (44.1)	0.108
Unexplained syncope	65 (23.8)	31 (24.2)	34 (23.5)	0.888
h/o SCD or sustained VT	7 (2.6)	3 (2.3)	4 (2.8)	1.000
Hypotension on ETT	33 (12.1)	16 (12.5)	17 (11.7)	0.855
Rest gradient >30mmHg	68 (24.9)	34 (26.6)	34 (23.5)	0.577
History of NSVT	78 (28.6)	32 (25.0)	46 (31.7)	0.230
Medications				
Beta-blocker	137 (50.2)	66 (51.6)	71 (49.0)	0.706
Anti-arrhythmic therapy	31 (11.3)	11 (8.6)	20 (13.8)	0.447

LV–left ventricle, LVOT–left ventricular outflow tract, NSVT–Non-sustained ventricular tachycardia, NYHA–New York Heart Association, SCD–Sudden Cardiac Death, VT–ventricular tachycardia.

### Population phenotypes

CMR characteristics for patients with and without LGE are shown in Table 2. Patients with LGE had a higher maximal wall thickness compared to patients without (median 20 vs 16, p<0.001). Patients with LGE also had lower LVEF (median 61.1 vs 65.5, p<0.001). Lastly, patients with LGE were less likely to have proximal septal hypertrophy (13.8% vs 39.8%, p<0.001) and more likely to have reverse curvature (44.8% vs 27.3%, p = 0.004) morphology.

**Table 2:** CMR characteristics stratified by late gadolinium enhancement.

	Total (n = 273)	No LGE (n = 128)	LGE+ (n = 145)	p-value
**LV mass indexed (g/m2)**	**77.3 (62.5–96.9)**	**69.1 (55.9–85.2)**	**87.7 (69.6–106.8)**	**<0.001**
**Maximal LV wall thickness (mm)**	**18 (16–21)**	**16 (15–19)**	**20 (16–24)**	**<0.001**
**LVEF (%)**	**63.8 (57.1–70.0)**	**65.5 (61.2–72.2)**	**61.1 (56.6–68.0)**	**<0.001**
LVEDVI (ml/m2)	82.0 (72.0–94.3)	80.0 (69.1–91.4)	83.3 (72.2–95.0)	0.063
**LVESVI (ml/m2)**	**29.3 (22.1–37.9)**	**26.9 (20.5–36.4)**	**31.9 (24.0–40.0)**	**0.002**
RVEF (%)	62.0 (56.6–67.7)	61.1 (55.2–67.8)	62.6 (58.7–67.6)	0.062
RVEDV indexed (ml/m2)	76.5 (62.3–89.1)	79.4 (62.7–90.9)	75.4 (62.0–88.5)	0.246
**RVESV indexed (ml/m2)**	**28.0 (20.8–36.9)**	**30.3 (21.5–39.1)**	**25.9 (20.5–35.2)**	**0.044**
Morphology				
**Sigmoid**	**71 (26.0)**	**51 (39.8)**	**20 (13.8)**	**<0.001**
**Reverse Curvature**	**100 (36.6)**	**35 (27.3)**	**65 (44.8)**	**0.004**
Apical	46 (16.9)	18 (14.1)	28 (19.3)	0.262
Concentric or indeterminate	56 (20.5)	24 (18.8)	32 (22.1)	0.550

EF–Ejection Fraction, EDV–End diastolic volume index, ESVI–End systolic volume index, LV–left ventricle, RV–right ventricle, SVI–stroke volume.

**Fig 2:**
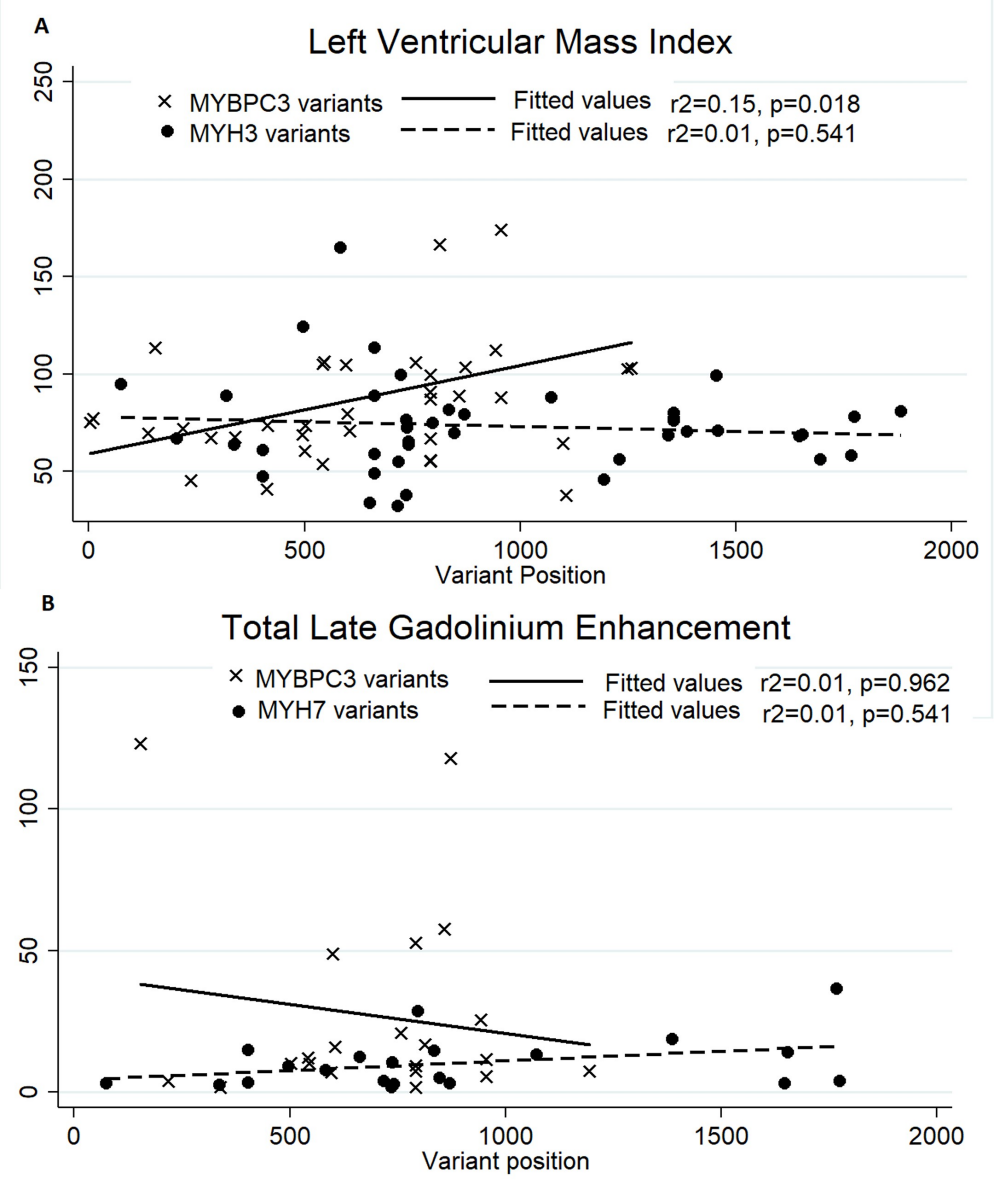
Correlation between variant position and phenotype. Panel A shows the correlation between variant position and left ventricular mass index. Variant position was associated with LVMI in *MYBPC3* variants (p = 0.018). Panel B shows the lack of correlation between variant position of total late gadolinium enhancement.

Genetic testing was performed in 202 patients. The CMR characteristics stratified by genetic testing diagnosis are shown in Table 3. Patients with pathogenic, likely pathogenic or rare *MYH7* variants had higher LVEF than those with *MYBPC3* variants (68.8 vs 59.1, p<0.001) and higher RVEF (67.3 vs 60.8, p = 0.018). Additionally, patients with *MYBPC3* variants were more likely to have LVEF < 55% (29.7% vs 4.9%, p = 0.005). However, maximal wall thickness, LV morphology, and presence of LGE were similar. Patients without an identifiable gene variant had higher LVMI (84.4 vs 72.2, p = 0.008). There was no difference in the proportion of patients with LGE between patients with and without identified gene variants (55.3% vs 44.4% p = 0.159). However, in those patients with LGE, scar volume was higher in patients with an identified pathogenic, likely pathogenic or rare variant (total scar 9.14 g vs 4.40 g, p = 0.020) and there was a higher proportion of LV mass replaced by scar (5.05% vs 2.28%, p = 0.002). Correlation between variant position and phenotype is shown in Fig 2. *MYBPC3* variant position was associated with LVMI (p = 0.018), but with poor overall correlation (r^2^ = 0.15).

**Table 3:** CMR characteristics stratified by genotype.

	*MYH7* (n = 41)	*MYBPC3* (n = 37)	Other gene variants (n = 12)	VUS (n = 13)	No identified mutation (n = 99)	*p* Value (*MYH7* vs *MYBPC3*)	p-value (No variant vs. any variant)
LVMI (g/m2)	70.4 (58.6–80.8)	77.2 (67.3–103.3)	68.8 (55.4–96.9)	72.1 (61.5–83.0)	84.4 (68.7–102.2)	0.066	**0.008**
Maximal LV thickness (mm)	18 (15–21)	20 (16–24)	19 (16–23)	18 (15–23)	18 (16–22)	0.075	0.486
LVEF (%)	68.8 (63.0–74.3)	59.1 (54.0–67.1)	64.0 (61.1–70.3)	61.6 (58.4–62.5)	66.4 (61.0–72.6)	**<0.001**	0.076
LVEDVI (ml/m2)	79.4 (69.0–94.0)	83.5 (76.4–98.3)	76.9 (69.5–92.1)	82.9 (77.2–99.5)	81.0 (70.6–94.7)	0.206	0.611
LVESVI (ml/m2)	23.9 (19.5–33.3)	32.1 (25.2–43.3)	25.9 (23.6–35.1)	32.3 (30.3–36.9)	26.3 (20.5–36.4)	**0.014**	0.092
RVEF (%)	67.3 (58.7–73.1)	60.8 (55.7–65.2)	61.6 (59.3–67.5)	59.9 (54.8–62.9	61.9 (55.9–69.2)	**0.018**	0.609
RVEDVI (ml/m2)	73.1 (57.2–86.9)	78.3 (64.2–91.4)	71.9 (62.5–96.9)	80.0 (67.1–89.8)	74.2 (60.5–88.9)	0.240	0.677
RVESVI (ml/m2)	23.8 (18.2–31.0)	31.4 (23.1–38.6)	28.3 (19.4–37.5)	33.8 (19.9–38.1)	27.8 (19.3–36.3)	0.022	0.973
Morphology							
Sigmoid	11 (26.8)	10 (26.8)	3 (25.0)	3 (23.1)	31 (31.3)	1.000	0.441
Reverse Curvature	18 (43.9)	21 (56.8)	4 (33.3)	3 (23.1)	28 (28.3)	0.365	**0.019**
Apical	7(17.1)	3 (8.1)	2 (16.7)	3 (23.1)	24 (24.2)	0.317	0.108
Concentric or Indeterminate	5 (12.2)	3 (8.1)	3 (25.0)	4 (30.8)	16 (16.2)	0.715	0.846
Any LGE	22 (53.7)	21 (56.8)	8 (66.7)	6 (46.2)	44 (44.4)	0.823	0.159
Any sub-endocardial	11 (26.8)	8 (21.6)	1 (8.3)	2 (15.4)	21 (21.2)	0.610	1.000
Any mid-myocardial	17 (41.5)	17 (46.0)	8 (66.7)	3 (23.1)	31 (31.3)	0.820	0.082
Any epicardial	6 (14.6)	7 (18.9)	2 (16.7)	1 (7.7)	8 (8.1)	0.763	0.129
LGE >50% wall thickness	12 (29.3)	7 (18.9)	0 (0.0)	1 (7.7)	13 (13.1)	0.307	0.257
LGE Segments	1 (0–5)	2 (0–5)	2 (0–4)	0 (0–4)	0 (0–3)	0.725	0.0252
Core Scar (g)	1.77 (1.06–5.03)	4.17 (1.92–9.14)	2.82 (0.59–5.46)	2.24 (0.85–7.52)	1.34 (0.33–4.59)	0.055	**0.019**
Gray Zone Scar (g)	6.40 (2.18–8.70)	7.88 (4.85–22.73)	3.08 (2.03–5.27)	5.46 (2.68–7.74)	2.72 (1.48–6.69)	0.114	**0.005**
Total scar (g)	8.32 (3.00–13.95)	11.49 (7.21–25.31)	6.01 (2.61–11.05)	7.69 (3.53–19.29)	4.40 (1.88–10.54)	0.099	**0.020**
Total Scar (%LV mass)	3.85 (2.37–10.65)	5.80 (3.39–13.20)	6.43 (2.08–8.90)	3.48 (1.70–14.79)	2.28 (1.02–6.73)	0.308	**0.002**

Core scar and gray zone scar were determined using the full-width, half-maximum method. Scar quantification reflects values in patients with visual LGE. EF–Ejection Fraction, EDVI–End diastolic volume index, ESVI–End systolic volume index, LGE–late gadolinium enhancement, LV–left ventricle, LVMI–left ventricular mass index, RV–right ventricle, SV–stroke volume, VUS–variant of uncertain significance.

### Clinical outcomes

Patients were followed clinically with median duration of follow-up of 1138 days (Interquartile range 230–1971). Clinical events during follow-up are shown in Table 4. Patients with LGE were more likely to have an ICD implanted (34.5 vs 19.5%, p = 0.007). However, there was no difference in the number of patients with an appropriate ICD shock (14% vs 16%, p = 1.000). Patients with LGE were more likely to have sustained VT or appropriate ICD shock (31.0 vs 19.5%, p = 0.037). Summary of multivariable Cox proportional Hazards analyses for the presence of LGE, adjusted for age and gender, are shown in S1 Fig. Presence of LGE was associated with increase in sustained VT or appropriate ICD shock (adjusted HR 1.94, 95% CI 1.16–3.24). However, LGE was not associated with a need for septal reduction therapy (adjusted HR 0.84, 95% CI 0.49–1.44, p = 0.53) or death (adjusted HR 1.12, 95% CI 0.33–3.86, p = 0.86). Similarly total scar volume was associated with an increase in sustained VT or appropriate ICD shock (adjusted HR 1.02 per g, 95% CI 1.00–1.03, p = 0.018), but not septal reduction therapy (adjusted HR 0.99, 95% CI 0.97–1.02) or death (adjusted HR 1.01, 95% CI 0.99–1.04, p = 0.343).

**Table 4:** Clinical outcomes stratified by presence of LGE.

	Total (n = 273)	No LGE (n = 128)	LGE+ (n = 145)	p-value
Myectomy/ Septal Ablation	56 (20.5)	31 (24.2)	25 (17.2)	0.177
**ICD Implanted**	**75 (27.5)**	**25 (19.5)**	**50 (34.5)**	**0.007**
Appropriate ICD Shock	11 of 75 (14.7)	4 of 25 (16.0)	7 of 50 (14.0)	1.000
**Sustained VT or Appropriate ICD shock**	**70 (25.6)**	**45 (31.0)**	**25 (19.5)**	**0.037**
Sudden Cardiac Death	6 (2.2)	4 (3.1)	2 (1.4)	0.424
All-cause mortality	11 (4.0)	6 (4.7)	5 (3.5)	0.760

ICD–implantable cardioverter defibrillator, VT–ventricular tachycardia.

**Fig 3:**
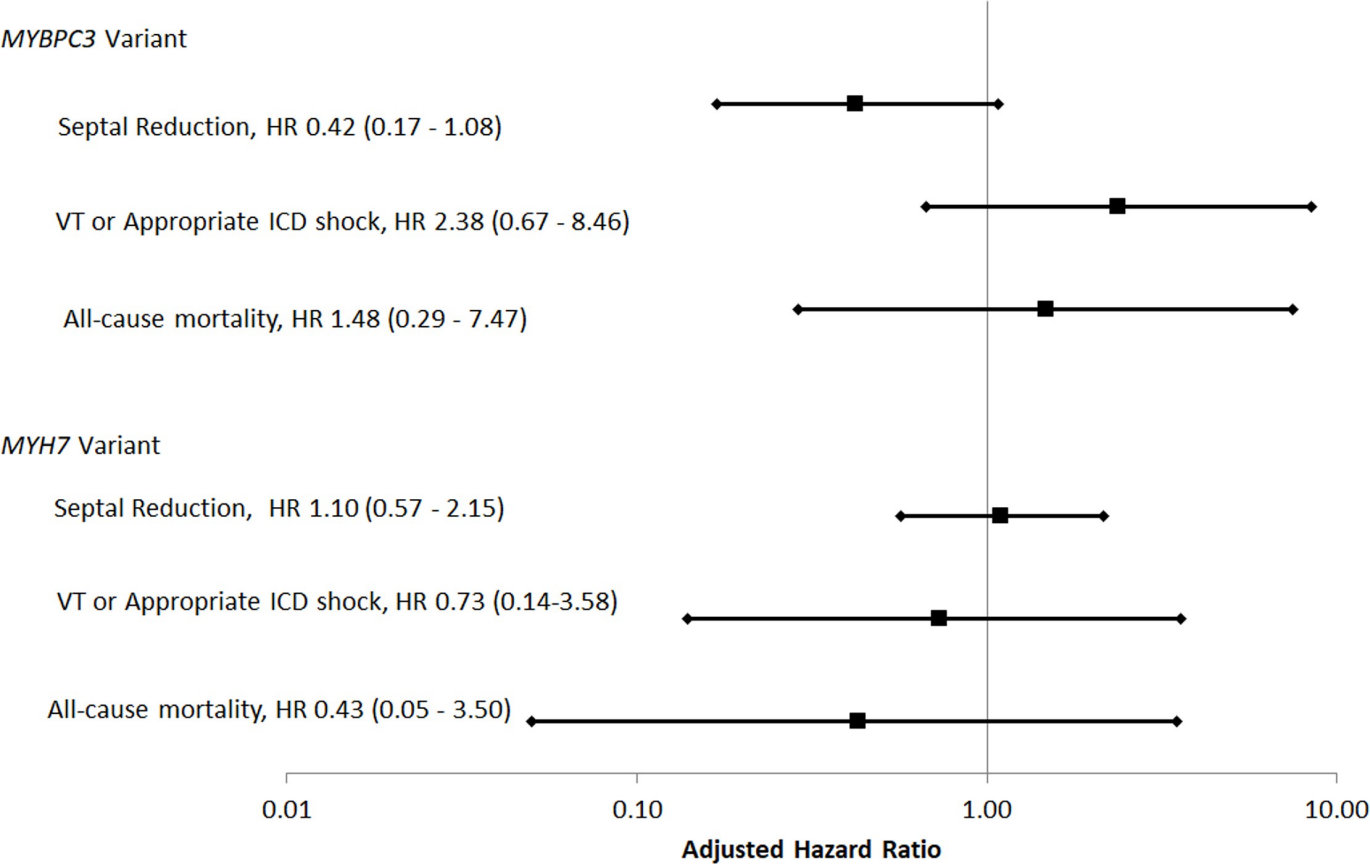
Summary of associations between clinical outcomes and genetic diagnosis. Note logarithmic scale.

Clinical events stratified by genetic testing findings are shown in Table 5. Patients with *MYBPC3* variants were more likely to have an ICD implanted than those with *MYH7* variants (54.1% vs 26.8%, p = 0.020) without a difference in appropriate ICD shock (4/20 [20.0%] vs 2 of 11 [18.2%], p = 1.00). There was a trend towards an increase in sustained VT or appropriate ICD shock in patients with *MYBPC3* variants (27.0% vs 12.2%, p = 0.150) Summary of multivariable Cox proportional hazards analyses, adjusted for age and gender, are shown in Fig 3. There was no difference in time to septal reduction therapy in patients with *MYBPC3* (adjusted HR 0.42, 95% CI 0.17–1.08, p = 0.071) or *MYH7* variants (adjusted HR 1.10, 95% CI 0.57–2.15, p = 0.768). Similarly, there was no difference in time to sustained VT or appropriate ICD shock with *MYBPC3* (adjusted HR 2.38, 95% CI 0.67–8.46, p = 0.1829) or *MYH7* gene variants (adjusted HR 0.73, 95% CI 0.14–3.58, p = 0.699). There was no increased risk of death with *MYBPC3* (1.48, 95% CI 0.29–7.47, p = 0.6329) or *MYH7* variants (0.43, 95% CI 0.05–3.50, p = 0.428).

**Table 5:** Clinical outcomes by genetic testing result.

	*MYH7* (n = 41)	*MYBPC3* (n = 37)	Other gene variants (n = 12)	VUS (n = 13)	No identified mutation (n = 99)	*p* Value (*MYH7* vs *MYBPC3*)	p-value (No variant vs. any variant)
Myectomy/ Septal Ablation	12 (29.3)	5 (13.5)	1 (8.3)	2 (15.4)	30 (30.3)	0.108	0.102
ICD Implanted	11 (26.8)	20 (54.1)	5 (41.7)	4 (30.8)	26 (26.3)	**0.020**	0.072
Appropriate ICD Shock	2 of 11 (18.2)	4 of 20 (20.0)	0 of 5 (0.0)	0 of 4 (0.0)	5 of 26 (19.2)	1.000	0.741
Sustained VT or Appropriate ICD shock	5 (12.2)	10 (27.0)	1 (8.3)	3 (23.1)	27 (27.3)	0.150	0.179
Sudden Cardiac Death	0 (0.0)	2 (5.4)	0 (0.0)	1 (7.7)	2 (2.0)	0.222	1.000
All-cause Mortality	1 (2.4)	2 (5.4)	0 (0.0)	0 (0.0)	6 (6.1)	0.601	0.324

ICD–implantable cardioverter defibrillator, VT–ventricular tachycardia.

## Discussion

Classically HCM has been characterized by poor correlation between genotype and phenotype. We sought to establish a correlation using CMR to characterize morphology which has potential benefits over echocardiography for this purpose. The high temporal and spatial resolution of CMR with superior intrinsic contrast allows more precise evaluation of myocardial morphology and reproducible quantitative assessment of ventricular volumes and function. [26–29] We found that patients with *MYBPC3* variants were more likely to have impaired ventricular function compared to patients with *MYH7* variants and had a trend towards an increase in arrhythmic events, with a higher proportion of patients receiving ICDs. Finally, we found that LGE burden was higher in patients with identifiable gene variants. Our findings, in a small population, suggest that there is correlation between genotype and phenotype, however the impact on clinical outcomes is less clear.

### Phenotype in patients with genetic variants

Genetic testing for HCM is used clinically in the form of targeted exonic sequencing of known disease-causing genes.[23] Echocardiography has traditionally been used for genotype-phenotype correlation in HCM, and prior studies have shown that the reverse curvature septal morphological subtype was a predictor of positive genetic testing.[30] Studies using CMR to help characterize HCM genotype-phenotype relationships have also found that more patients with any genetic mutation had reverse curvature HCM in comparison to sigmoidal HCM or apical HCM, indicating that CMR may be useful in genotype-phenotype analysis.[19] In the same study, it was noted that LGE was more common in those with a positive genetic test in comparison to those with a negative test. In our study, we found no association between presence of LGE and genetic diagnosis but did find larger volumes of LGE associated with the presence of an identifiable variant. Additionally, we did not see an association between genetic testing result and overall morphology. We used contemporary genetic testing data and found a larger proportion of patients with abnormal genetic testing compared to previous studies.[30,31]

### Differences between *MYH7* and *MYBPC3*

In our cohort, the most common variants occurred in *MYH7* and *MYBPC3*. Our analysis revealed that the presence of a *MYBPC3* variant was associated with a lower LVEF and a higher prevalence of low LVEF. Interestingly, Weissler-Snir et al. found a similar trend in a cohort of HCM patients characterized with CMR.[31] No other mutation groups were sufficiently prevalent to allow further characterization. *MYBPC3* is a key component of myocardial thick filaments and variants have been associated with dilated cardiomyopathy.[32] Additionally, Additionally, *MYBPC3* variants have been associated with impaired ventricular function in patients with coronary artery disease.[33] However, it is not clear why ventricular function is less impaired in patients with *MYH7* variants since an interaction between the two genes seems to be necessary to maintain systolic function.[34] There was also a trend towards increased LGE burden in patients with *MYBPC3* variants, which itself was associated with an increase in sustained VT or ICD shock. While we did not see these phenotypic differences translate into clinical outcomes, differences may be seen in larger populations. Data from the sarcomeric human cardiomyopathy (SHARE) registry showed that overall clinical outcomes may be worse in patients with *MYH7* variants.[35] However, they found that the incidence of cardiac arrest was higher in patients with *MYBPC3* variants.[35] Interestingly, variant position was significantly associated with LVMI in patients with *MYBPC3* variants, although with poor overall correlation. Our findings suggest that wider use of CMR as well as genetic testing may help to characterize the phenotypes of other disease-associated genes.

### Correlation between CMR characteristics and clinical outcomes

One of the major benefits of CMR characterization in patients with HCM is to quantify LGE which has significant prognostic ability.[16,17] Our findings were consistent with previously published data that demonstrated increased LGE in HCM particularly in areas of increased wall thickness,[16]. and that reverse curvature septal morphology was associated with LGE as previously described.[19,36] We found ICD implantation to be more common, likely representing this increased incidence as well as the use of LGE as a risk stratification tool.[37] ICD shocks were not more common in this group compared to patients without LGE. However, given the low incidence of events in this population and the importance of LGE in other larger cohorts it is likely that our study was not sufficiently powered to demonstrate an association.[38] [39,40]

### Limitations

Our study has several important limitations. We had a relatively small patient sample and were not able to assess phenotypic features seen in less frequent gene variants. Since the presence of LGE was based on presence on two orthogonal views, it’s possible that small foci of LGE were not included. Additionally, variation in gadolinium dosing may have impacted LGE identification and quantification. Some of our negative findings, particularly with respect to LGE, may be due to lack of statistical power. However, we were able to demonstrate differences in morphology between the most common variants. We did not assess RV mass, RV wall thickness, or atrial morphology and these may also be associated with genotype. The small patient sample, with limited follow-up duration in some patients, may have impaired our ability to assess for differences in clinical outcomes between groups. However, our data may provide mechanistic insights into data from larger studies such as the SHARE registry. Finally, genetic testing was not complete in our cohort which may have influenced some of our findings. However, our study is one of the largest published cohorts with comprehensive CMR and genetic characterization to date.

## Conclusions

CMR may be useful to characterize genotype-phenotype relationships in HCM. We found that patients with *MYBPC3* mutations were more likely to have impaired ventricular function and may be more prone to arrhythmic events. Larger studies using CMR phenotyping may be capable of identifying additional characteristics associated with less frequent genetic causes of HCM.

## Supporting information

S1 Fig Summary of associations between clinical outcomes and presence of LGE. HR–hazard ratio.
(TIF)

S1 Table Classification of genetic variants. Several genetic variants were present in more than one patient and several patients had more than one genetic variant.
(DOCX)

S2 Table CMR characteristics in sensitivity analysis. * indicates statistically significant difference between VUS and other groups. EF–Ejection Fraction, EDV–End diastolic volume, ESV–End systolic volume, LV–left ventricle, LVMI–Left ventricular mass index, RV–right ventricle, SV–stroke volume.
(DOCX)

S3 Table Clinical outcomes stratified by genetic testing diagnosis in sensitivity analysis. ICD–implantable cardioverter defibrillator, VT–ventricular tachycardia.
(DOCX)

S4 Table Baseline population characteristics by genetic testing referral. LV–left ventricle, LVOT–left ventricular outflow tract, NSVT–Non-sustained ventricular tachycardia, NYHA–New York Heart Association, SCD–Sudden Cardiac Death, VT–ventricular tachycardia.
(DOCX)

## Abbreviations

CHF: congestive heart failure
CMR: cardiac magnetic resonance
HCM: hypertrophic cardiomyopathy
ICD: Implanted cardiac defibrillator
IQR: interquartile range
LV: left ventricle
LVEDV: Left ventricular end-diastolic volume
LVEF: left ventricular ejection fraction
LVESV: Left ventricular end-systolic volume
LVMI: left ventricular mass index
LGE: late gadolinium enhancement
MYH7: beta-myosin heavy chain
MYBPC3: cardiac myosin binding protein C
NYHA: New York Heart Association
RVEF: right ventricular ejection fraction
RVEDV: right ventricular end-diastolic volume
RVESV: right ventricular end-systolic volume
SCD: sudden cardiac death
SD: standard deviation
VUS: variant of uncertain significance
