# Supplementary figures and images for: Defining genotype-phenotype relationships in patients with hypertrophic cardiomyopathy using cardiovascular magnetic resonance imaging
Source: PLoS One. 2019 Jun 14;14(6):e0217612. doi: 10.1371/journal.pone.0217612 (PMC6568393; doi:10.1371/journal.pone.0217612)

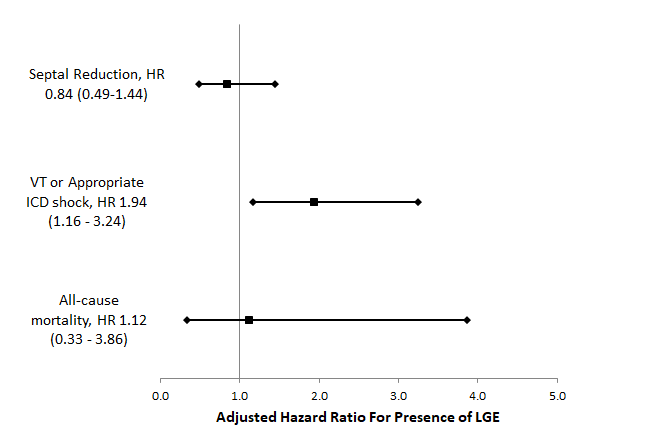

Supplement: S1 Fig — HR–hazard ratio. (TIF) [file pone.0217612.s001.tif]
