# Supplementary material for: Defining genotype-phenotype relationships in patients with hypertrophic cardiomyopathy using cardiovascular magnetic resonance imaging
Source: PLoS One. 2019 Jun 14;14(6):e0217612. doi: 10.1371/journal.pone.0217612 (PMC6568393; doi:10.1371/journal.pone.0217612)
Supplement: S1 Table — Several genetic variants were present in more than one patient and several patients had more than one genetic variant. (DOCX) [file pone.0217612.s002.docx]

**S1 Table. Classification of genetic variants**.

| **Gene** | **Disease Causing Variants** | **Variants of Uncertain Significance** |
| --- | --- | --- |
| *ACTN2* |  | ACTN2 p.Thr716Met, ACTN2 exons 8-10 deletion |
| *ALMS1* |  | ALMS1 p.Glu200Gly, ALMS1 p.Ser524_Leu525insPro, ALMS1 p.Arg3805Thr, ALMS1 p.Ser3954Ala, |
| *FLNC* |  | FLNC p.Glu200Gly |
| *JPH2* |  | JPH2 p.Arg436Cys |
| *KCNH2* |  | KCNH2 p.Arg1033Gln |
| *MYBPC3* | MYBPC3 p.Ser137Ter, MYBPC3 p.Ile154LeufsX5, MYBPC3 p.Val219Leu, MYBPC3 p.Gln339Ter, MYBPC3 p.Arg496Gln, MYBPC3 p.Arg502Trp, MYBPC3 p.Glu542Gln, MYBPC3 p.Thr737Met, MYBPC3 p.Trp792Arg, MYBPC3 p.Trp792ValfsX41, MYBPC3 p.Arg943Ter, MYBPC3 p.Pro955ArgfsX95, MYBPC3 p.Arg1073ProfsX4, MYBPC3 p.Tyr1100ValfsX49, MYBPC3 p.Gly1195Val, MYBPC3 p.Gly1248_Cys1253dup, MYBPC3 p.Asn1257Lys, MYBPC3 IVS11-2 A>g, MYBPC3 IVS14-2 A>G, MYBPC3 IVS17+4A>T, MYBPC3 IVS30+2T>G, | MYBPC3 p.Tyr237Ser, MYBPC3 p.Ile284Val, MYBPC3 p.Glu413Gly, MYBPC3 p.Gly596Arg, MYBPC3 p.Lys600AsnfsX2, MYBPC3 p.Lys812Arg, MYBPC3 p.Ser858AsnMYBPC3 p.Pro873His, MYBPC3 p.Ala1194Val, |
| *MYH7* | MYH7 p.Gly74Arg, MYH7 p.Arg169Gly, MYH7 p.Arg204His, MYH7 p.Val320Met, MYH7 p.Val338Met, MYH7 p.Arg403Gln, MYH7 p.Glu497Asp, MYH7 p.Gly584Arg, MYH7 p.Arg652Gly, MYH7 p.Arg663His, MYH7 p.Gly716Arg, MYH7 p.Arg719Trp, MYH7 p.Arg723Cys, MYH7 p.Ile736Thr, MYH7 p.Gly741Arg, MYH7 p.Ala797Thr, MYH7 p.Lys847del, MYH7 p.Arg1344Gln, MYH7 p.Glu1356Lys, MYH7 p.Lys1459Asn, MYH7 p.Glu1468Lys | MYH7 p.Gly636Ser, MYH7 p.Ser738Thr, MYH7 p.Ala742Thr, MYH7 p.Arg787His, MYH7 p.Ile836Met, , MYH7 p.Arg1136His, MYH7 p.Val1213Met, MYH7 p.Glu1387Lys, MYH7 p.Glu1455Lys, MYH7 p.Arg1560Gln, MYH7 p.Glu1768Lys, MYH7 p.Ser1776Gly, MYH7 p.Glu1883Lys, MYH7 IVS32+1 G>C |
| *MYL2* | MYL2 p.Arg58Gln | MYL2 p.Met1? |
| *MYL3* |  | MYL3 p.Glu143Lys |
| *PRDM16* |  | PRDM16 p.Asp1125_Asp1130del |
| *TNNI3* | TNNI3 c.25-8T>A, TNNI3 p.Arg145Trp, TNNI3 p.Ser166Phe, | TNNI3 p.Arg79Cys |
| *TNNT2* | TNNT2 p.Arg92Gln, TNNT2 p.Lys97Asn, TNNT2 IVS11-1G>A, |  |
| *TPM1* | TPM1 p.Ala22Thr | TPM1 p.Glu23Asp, TPM1 p.Lys66Asn, |
| *TTN* |  | TTN c.53543T>C, TTN c.77564G>A, TTN p.Gly5457Ser |

**S1 Table. Classification of genetic variants.** Several genetic variants were present in more than one patient and several patients had more than one genetic variant.
