# Supplementary material for: Defining genotype-phenotype relationships in patients with hypertrophic cardiomyopathy using cardiovascular magnetic resonance imaging
Source: PLoS One. 2019 Jun 14;14(6):e0217612. doi: 10.1371/journal.pone.0217612 (PMC6568393; doi:10.1371/journal.pone.0217612)
Supplement: S2 Table — * indicates statistically significant difference between VUS and other groups. EF–Ejection Fraction, EDV–End diastolic volume, ESV–End systolic volume, LV–left ventricle, LVMI–Left ventricular mass index, RV–right ventricle, SV–stroke volume. (DOCX) [file pone.0217612.s003.docx]

**Supplemental Table 2. CMR Characteristics in sensitivity analysis**

|  | *MYH7*  (n=30) | *MYBPC3*  (n=29) | Other gene variants (n=12) | VUS  (n=32) | No identified mutation  (n=99) | *p* Value (MYHC vs MYBPC) | p-value  (No mutation vs. any mutation) |
| --- | --- | --- | --- | --- | --- | --- | --- |
| LVMI (g/m2) | 69.1 (58.6 – 87.7) | 77.2 (67.6 – 104.8) | 68.8 (55.4 – 96.9) | 72.3 (59.8 – 82.3) | 84.4 (68.7 – 102.2) | 0.066 | **0.008** |
| Maximal LV wall thickness | 18 (16 – 21) | 20 (17 – 24) | 19 (16 – 23) | 17 (15 – 21) | 18 (16- 22) | 0.144 | 0.486 |
| LVEF (%) | 68.4 (63.0 – 74.3) | 59.1 (54.0 – 67.1) | 64.0 (61.1 – 70.3) | 62.3 (57.7 – 70.4) | 66.4 (61.0 – 72.6) | **0.005** | 0.076 |
| LVEDV indexed (ml/m2) | 77.2 (68.0– 102.0) | 84.7 (76.4 – 98.8) | 76.9 (69.5 – 92.1) | 81.7 (73.9 – 89.7) | 81.0 (70.6 – 94.7) | 0.296 | 0.611 |
| LVESV indexed (ml/m2) | 24.2 (19.1 – 42.0) | 32.1 (25.2 – 42.8) | 25.9 (23.6 – 35.1) | 28.6 (22.4 – 36.3) | 26.3 (20.5– 36.4) | 0.122 | 0.092 |
| RVEF (%) | 66.1 (57.9 – 72.7) | 62.3 (58.0 – 67.2) | 61.6 (59.3 – 67.5) | 60.5 (55.5 – 69.4) | 61.9 (55.9 – 69.2) | 0.163 | 0.609 |
| RVEDV indexed (ml/m2) | 75.9 (57.2 – 87.1) | 78.3 (67.1 – 92.5) | 71.9 (62.5 – 96.9) | 77.5 (62.4 – 87.9) | 74.2 (60.5 – 88.9) | 0.388 | 0.677 |
| RVESV indexed (ml/m2) | 23.9 (18.2 – 35.0) | 31.3 (24.3 – 37.0) | 28.3 (19.4 – 37.5) | 25.5 (19.8 – 37.7) | 27.8 (19.3 – 36.3) | 0.154 | 0.973 |
| Morphology |  |  |  |  |  |  |  |
| Sigmoid | 8 (26.7) | 8 (27.6) | 3 (25.0) | 8 (25.0) | 31 (31.3) | 1.000 | 0.441 |
| Reverse Curvature | 17 (56.7) | 19 (65.5) | 4 (33.3) | **6 (18.8)*** | 28 (28.3) | 0.596 | 0.019 |
| Apical | 2 (6.7) | 0 (0.0) | 2 (16.7) | **11 (34.4)*** | 24 (24.2) | 0.492 | 0.108 |
| Concentric or Indeterminate | 3 (10.0) | 2 (6.9) | 3 (25.0) | 7 (21.9) | 16 (16.2) | 1.000 | 0.846 |
| Any LGE | 15 (53.5) | 17 (53.5) | 8 (66.7) | 17 (53.1) | 44 (44.4) | 1.000 | 0.159 |
| Any sub-endocardial | 7 (24.1) | 6 (20.0) | 1 (8.3) | 8 (25.0) | 21 (21.2) | 0.761 | 1.000 |
| Any mid-myocardial | 11 (36.7) | 15 (51.7) | 8 (66.7) | 11 (34.4) | 31 (31.3) | 0.299 | 0.082 |
| Any epicardial | 2 (6.7) | 5 (17.2) | 2 (16.7) | 7 (21.9) | 8 (8.1) | 0.254 | 0.129 |
| LGE >50% wall thickness | 7 (23.3) | 5 (17.2) | 0 (0.0) | 8 (25.0) | 13 (13.1) | 0.748 | 0.257 |
| LGE Segments | 1 (0 – 4) | 2 (0 – 5) | 2 (0 – 4) | 1 (0 – 5) | 0 (0 – 3) | 0.609 | 0.0252 |
| Core Scar (g) | 1.37 (10.71 – 4.08) | 3.61 (1.91 – 5.6) | 2.82 (0.59 – 5.46) | 3.74 (1.90 – 11.55) | 1.34 (0.33 – 4.59) | 0.067 | **0.019** |
| Gray Zone Scar (g) | 3.35 (2.18 – 8.06) | 7.30 (4.13 – 11.10) | 3.08 (2.03 – 5.27) | 7.74 (4.85 – 13.49) | 2.72 (1.48 – 6.69) | 0.168 | **0.005** |
| Total scar (g) | 4.89 (2.92 – 12.14) | 10.18 (7.21 – 16.7) | 6.01 (2.61 – 11.05) | 13.95 (6.77 – 20.90) | 4.40 (1.88 – 10.54) | 0.126 | **0.020** |
| Total Scar (% of LV mass) | 3.77 (2.28 – 7.40) | 5.04 (3.27 – 9.36) | 6.43 (2.08 – 8.90) | 10.65 (2.66 – 17.42) | 2.28 (1.02 – 6.73) | 0.249 | **0.002** |

**S2 Table. CMR Characteristics in sensitivity analysis.** Core scar and gray zone scar were determined using the full-width, half-maximum method. Scar quantification reflects values in patients with visual LGE * indicates statistically significant difference between VUS and other groups. EF – Ejection Fraction, EDV – End diastolic volume, ESV – End systolic volume, LV – left ventricle, LVMI – Left ventricular mass index, RV – right ventricle, SV – stroke volume.
