# Supplementary material for: Defining genotype-phenotype relationships in patients with hypertrophic cardiomyopathy using cardiovascular magnetic resonance imaging
Source: PLoS One. 2019 Jun 14;14(6):e0217612. doi: 10.1371/journal.pone.0217612 (PMC6568393; doi:10.1371/journal.pone.0217612)
Supplement: S3 Table — ICD–implantable cardioverter defibrillator, VT–ventricular tachycardia. (DOCX) [file pone.0217612.s004.docx]

**S3 Table. Clinical Outcomes in Sensitivity Analysis**

|  | *MYH7*  (n=30) | *MYBPC3*  (n=29) | Other gene variants (n=12) | VUS  (n=32) | No identified mutation  (n=99) | *p* Value (*MYH7* vs *MYBPC3*) | p-value  (No variant vs. any variant) |
| --- | --- | --- | --- | --- | --- | --- | --- |
| Myectomy/ Septal Ablation | 7 (23.3) | 5 (17.2) | 1 (8.3) | 7 (21.9) | 30 (30.3) | 0.748 | 0.139 |
| ICD Implanted | 8 (26.7) | 17 (58.6) | 5 (41.7) | 10 (31.3) | 26 (26.3) | 0.018 | 0.072 |
| ICD Shock | 2 of 8 (25.0) | 2 of 17 (11.8) | 0 of 5  (0.0) | 2 of 10 (20.0) | 5 of 26  (19.2) | 0.570 | 1.000 |
| Sustained VT or Appropriate ICD shock | 7 (24.1) | 5 (16.7) | 1 (8.3) | 6 (18.8) | 27 (27.3) | 0.532 | 0.179 |
| Sudden Cardiac Death | 0 (0.0) | 2 (6.9) | 0 (0.0) | 1 (3.1) | 2 (2.0) | 0.237 | 1.000 |
| All-Cause Mortality | 1 (3.3) | 1 (3.5) | 0 (0.0) | 1 (3.1) | 6 (6.1) | 1.000 | 0.511 |

**S3 Table. Clinical outcomes stratified by genetic testing diagnosis in sensitivity analysis**. ICD – implantable cardioverter defibrillator, VT – ventricular tachycardia.
