# Supplementary material for: Defining genotype-phenotype relationships in patients with hypertrophic cardiomyopathy using cardiovascular magnetic resonance imaging
Source: PLoS One. 2019 Jun 14;14(6):e0217612. doi: 10.1371/journal.pone.0217612 (PMC6568393; doi:10.1371/journal.pone.0217612)
Supplement: S4 Table — LV–left ventricle, LVOT–left ventricular outflow tract, NSVT–Non-sustained ventricular tachycardia, NYHA–New York Heart Association, SCD–Sudden Cardiac Death, VT–ventricular tachycardia. (DOCX) [file pone.0217612.s005.docx]

|  | Total  (n = 273) | No Genetic Testing  (n = 71) | Genetic Testing  (n = 202) | *p* Value |
| --- | --- | --- | --- | --- |
| Age at CMR | 51.2 ± 15.5 | 53.9 ± 14.9 | 50.3 ± 15.7 | 0.098 |
| Male (%) | 173 (62.9) | 44 (62.0) | 129 (63.9) | 0.776 |
| BSA (m2) | 1.94 ± 0.25 | 1.90 ± 0.26 | 1.96 ± 0.24 | 0.082 |
| Maximal LV wall thickness (mm) | 18 (16-21) | 18 (15 – 20) | 18 (16 – 22) | 0.082 |
| Ethnicity |  |  |  |  |
| Caucasian | 176 (64.5) | 42 (59.2) | 134 (66.3) | 0.314 |
| Asian | 41 (15.0) | 12 (16.9) | 29 (14.4) | 0.699 |
| Latino | 24 (8.8) | 5 (7.0) | 19 (9.4) | 0.634 |
| African-American | 9 (3.3) | 2 (2.8) | 7 (3.5) | 1.000 |
| Other | 22 (8.1) | 9 (12.7) | 13 (6.4) | 0.126 |
| Congestive Heart Failure |  |  |  |  |
| NYHA I | 163 (59.7) | 47 (66.2) | 116 (57.4) | 0.208 |
| NYHA II | 73 (26.7) | 12 (16.9) | 61 (30.2) | 0.030 |
| NYHA III | 34 (12.5) | 9 (12.7) | 25 (12.4) | 1.000 |
| NYHA IV | 4 (1.5) | 3 (4.2) | 1 (0.50) | 1.000 |
| Resting LVOT gradient | **2 (0 – 28)** | **0 (0 – 14)** | **7 (0 – 33)** | **0.009** |
| Atrial Fibrillation | **30 (11.0)** | **0 (0.0)** | **30 (14.9)** | **<0.001** |
| Dyslipidemia | 84 (30.8) | 17 (23.9) | 67 (33.2) | 0.179 |
| Hypertension | 108 (39.6) | 27 (38.0) | 81 (40.1) | 0.780 |
| Diabetes | 19 (7.0) | 7 (9.9) | 12 (5.9) | 0.282 |
| Risk Factors for SCD |  |  |  |  |
| LV wall thickness > 30 mm | 9 (3.3) | 2 92.8) | 7 (3.5) | 1.000 |
| FH SCD | 108 (39.6) | 25 (35.2) | 83 (41.1) | 0.401 |
| Unexplained syncope | 65 (23.8) | 12 (16.9) | 43 (21.3) | 0.651 |
| h/o SCD or sustained VT | 7 (2.6) | 3 (2.3) | 4 (2.8) | 1.000 |
| Hypotension on ETT | **33 (12.1)** | **0 (0.0)** | **33 (16.3)** | **<0.001** |
| Rest gradient >30mmHg | 68 (24.9) | 12 (16.9) | 56 (27.7) | 0.080 |
| History of NSVT | 78 (28.6) | 13 (18.3) | 65 (32.2) | 0.032 |
| Medications |  |  |  |  |
| Beta-blocker | 137 (50.2) | 35 (49.3) | 102 (50.5) | 0.891 |
| Anti-arrhythmic therapy | 31 (11.3) | 7 (9.9) | 24 (11.9) | 0.828 |

**S4 Table. Baseline Population Characteristics by Genetic Testing Referral.**

**S4 Table. Baseline Population Characteristics by genetic testing referral.** LV – left ventricle, LVOT – left ventricular outflow tract, NSVT – Non-sustained ventricular tachycardia, NYHA – New York Heart Association, SCD – Sudden Cardiac Death, VT – ventricular tachycardia.
